# Au Gratings Fabricated by Interference Lithography for Experimental Study of Localized and Propagating Surface Plasmons

**DOI:** 10.1186/s11671-017-1965-4

**Published:** 2017-03-11

**Authors:** Viktor Dan’ko, Mykola Dmitruk, Ivan Indutnyi, Sergiy Mamykin, Victor Myn’ko, Petro Shepeliavyi, Mariia Lukaniuk, Petro Lytvyn

**Affiliations:** grid.466789.2 V. Lashkaryov Institute of Semiconductor Physics, Nat. Acad. of Sci. of Ukraine, 45, Prospect Nauky, 03028 Kyiv, Ukraine

**Keywords:** Plasmonic gratings, Surface plasmon resonance, Interference lithography, 73.20 Mf, 85.40 Hp, 42.79.Dj

## Abstract

Optical properties of high-frequency Au gratings with a fixed period (296.6 ± 0.5 nm) and a variable modulation depth are studied using measurements of spectral and angular dependence of transmission and reflection of polarized light in order to build the dispersion curves of excited optical modes and to identify their types. It was shown that in gratings with small modulation depth only propagating surface plasmon-polaritons (SPP) modes were observed. With increasing of modulation depth, the intensity of SPP decreases and localized plasmon (LP) resonance appears, which is more intense at small incident angles, and overlaps with the SPP modes. For grating with isolated grooves (nanowires), mostly LP resonance is observed. After additional deposition of gold onto grating with isolated grooves, the intensity of the SPP mode increases again, and the LP band maximum shifts to longer wavelengths.

## Background

Plasmonic gratings that support both localized plasmon resonant oscillations and propagating surface plasmon-polaritons, electromagnetic waves coupled to electron density oscillations and propagating along the interface between metal and dielectric, have found wide use in recent years in numerous fields of research and applications. Such plasmonic structures are widely used in a variety of sensors based on plasmon resonance [1], in surface-enhanced Raman spectroscopy [2] and light harvesting [3, 4], as plasmon launchers [5], directional emitters [6], polarization converters [7], sub-wavelength optical elements [8], and others [9].

In the gratings with small relief depth, surface plasmons are propagating, i.e., neighboring grooves of the grating are coupled by the plasmonic wave traveling along the metal-dielectric interface. However, if the grating has a large depth of relief or consists of nanowires, increasing the slit width between nanowires eventually results in LP excitations in isolated nanostripes. Many studies have been devoted to the coupling between isolated plasmonic nanoobjects [10–12], including investigation of the transition between the regimes of LP and SPP in gold gratings with a fixed period and a variable filling factor [13, 14]. In the previous work [13], we have demonstrated experimentally that after thermal treatment of one-dimensional (gratings) and two-dimensional (arrays) periodic (period 500 nm) Au structures, nanowires and nanoislands became more compact structures due to “shrinkage”. As the result, the slit width between the edges of nanowires or nanodots rises up, and the intensity of SPP decreases, but LP intensity increases significantly. In another paper [14], authors can identify different plasmon excitation regimes depending on the slit width in thin gold nanoslit gratings with a fixed period of 750 ± 35 nm. Only localized plasmons are observed for the gratings with slits between the nanowires wider than approximately 0.7 resonant wavelengths. The localized plasmons begin to couple, and weak SPP excitation becomes possible as the slit width is reduced (transition regime). For more narrow slits, the transmittance spectra show features associated with pure SPP excitation.

The intermediate regime for gratings with a different depth of relief (when both localized and propagating plasmons are excited) is still investigated insufficiently both theoretically and experimentally. Difficulties in the experimental study of high-frequency gratings are connected primarily with the complexities of manufacturing samples with the specified characteristics. One of the most technological methods for fabrication of periodic nanostructures and microstructures is interference lithography (IL) [15]. Here, we report the IL technique with the use of vacuum chalcogenide glass resists for the formation of high-frequency gratings (period 296.6 ± 0.5 nm) on the surface of Au layers and detailed studies of the influence of grating relief depth on features of excited plasmons in such periodic structures.

## Methods

The samples for our experiments were prepared by successive thermal vacuum deposition of a 1–3-nm-thick (effective thickness) Cr adhesive layer, a layer of metal (Au) with a thickness of 40–50 nm, and a photoresist layer (As_40_S_40_Se_20_) with a thickness of around 100 nm onto polished glass substrates (dimensions: 20 × 20 × 1 mm) at a residual pressure of 2 × 10^−3^ Pa. The deposition rate and film thicknesses were monitored in situ with a calibrated KIT-1 quartz thickness meter.

The recording of periodic structures on photoresist films was carried out using the interference pattern formed with a helium-cadmium laser (wavelength λ = 441.6 nm) using the holographic setup assembled by the wave-amplitude division method. After exposure, the samples were chemically treated in non-water alkaline organic solutions to form a resistive mask in the photoresist layer, through which the metal film was etched. By varying the etching time of Au layer, it was possible to change the depth of the relief thus formed gratings. The Au grating structures with different depth of relief were obtained after removing the photoresist residues in alkaline solution, washing, and drying. The structured area of each sample was 18 × 18 mm^2^.

The surface patterns of the gratings were examined with a Dimension 3000 Scanning Probe atomic force microscope (Digital Instruments Inc., Tonawanda, NY, USA) in the atomic force microscope (AFM) tapping mode.

**Fig. 1:**
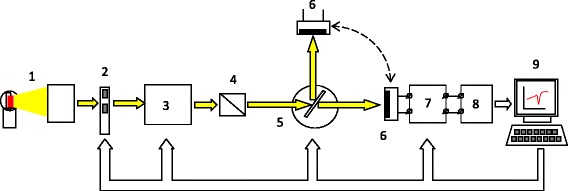
The scheme of experimental setup for the measurements of light reflectance *R* and transmittance *T*. (*1*) Illuminator. (*2*) Mechanical light chopper. (*3*) Monochromator. (*4*) Glan prism. (5) Rotary table for samples. (*6*) Silicon photodetector. (*7*) Amplifier and demodulator. (*8*) Analog-to-digital converter. (*9*) Personal computer

Optical properties of fabricated structures were studied using measurements of spectral and angular dependence of transmission and reflection of polarized light in the 0.4–1.1 μm wavelength range and 10–80° angles of incidence. The automated setup for the measurements consists of an illuminator, a mechanical light chopper, a monochromator with Glan prism at the exit, and a rotary table for samples (the scheme of setup is shown on Fig. 1). The intensity of the light reflection or transmission was measured by silicon photodetector, signal of which after amplification and demodulation was applied to the input of analog-to-digital converter. The measurements allow to build the dispersion curves of excited optical modes and to identify their types.

## Results and Discussion

**Fig. 2:**
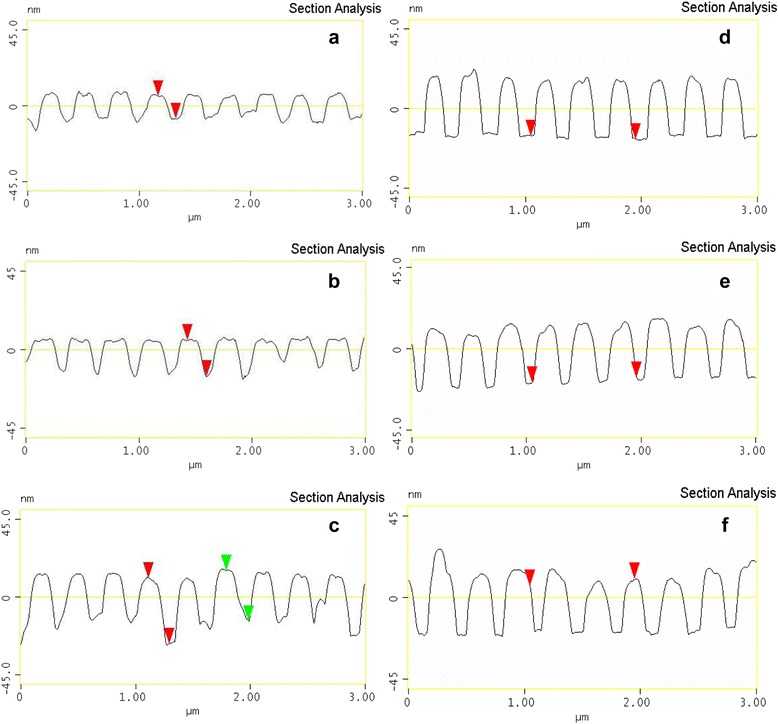
AFM cross-sections of Au gratings with a period of 296.6 ± 0.5 nm, modulation depth of 13.0 ± 3 nm (**a**), 20 ± 3 nm (**b**), 29.0 ± 3 nm (**c**), and 35.0 ± 3 nm (**d**), after additional deposition of 10 nm of Au (**e**) and 20 nm of Au (**f**)

Figure 2a–d shows cross-sections obtained from AFM data of four gratings with the same period of 296.6 ± 0.5 nm, but different time of Au etching through the photoresist mask resulting in different average modulation depth (groove depth) of the gratings: 13.0 ± 3 nm (a), 20 ± 3 nm (b), 29.0 ± 3 nm (c), and 35.0 ± 3 nm (d). The initial thickness of Au layer was about 40 nm, and with increasing time of etching, the metal layer thickness between the grating grooves is reduced. Grating (d) which was etched the longest time is essentially a periodic array of isolated Au nanowires that are not connected by a metal interlayer. To study the dependence of SPP and LP excitation on the thickness of Au interlayer between the grating grooves at the same depth of modulation, we additionally deposited 10 and 20 nm of Au onto (d) samples (Fig. 2e, f, respectively). As can be seen, the additional deposition of 10 and 20 nm of gold did not change the depth of modulation, which has remained equal to 35 nm, but the average half-width of the grooves slightly increased.

**Fig. 3:**
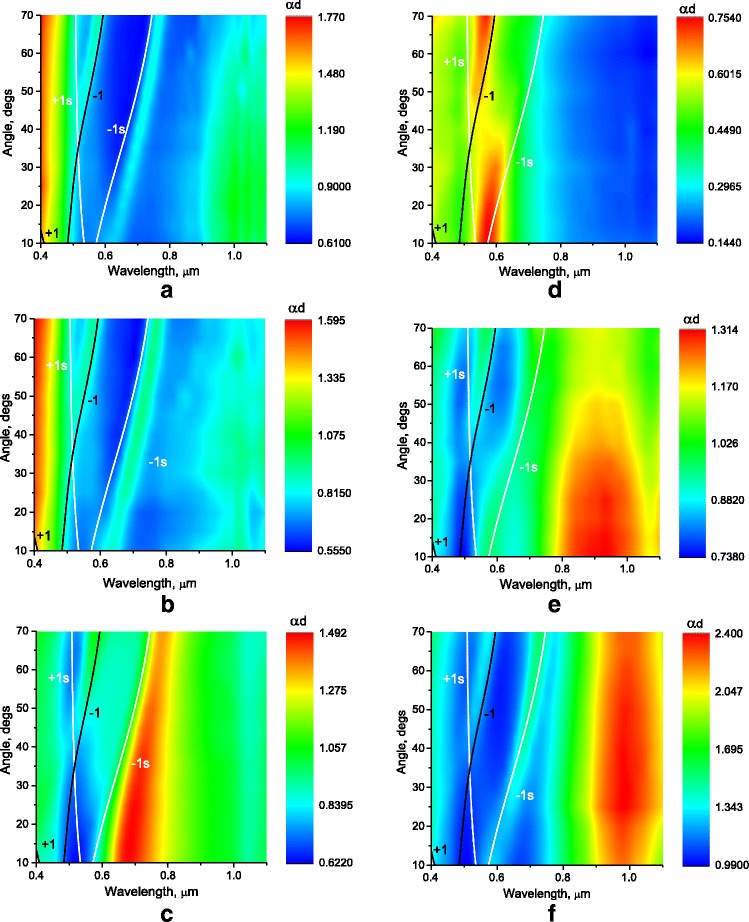
P-polarized extinction (αd) as a function of wavelength and angle of incidence on Au gratings with period of 296.6 ± 0.5 nm, modulation depth of 13.0 ± 3 nm (**a**), 20 ± 3 nm (**b**), 29.0 ± 3 nm (**c**), and 35.0 ± 3 nm (**d**), and after additional deposition of Au: 10 nm (**e**) and 20 nm (**f**) overlapped with the dispersion curves calculated from Eqs. 1 and 2. The dispersion curves of the surface plasmon-polaritons corresponding to the air-metal interface (1,−1) and substrate-metal interface (1s,−1s), for *m* = +1 (1,1s) and *m* = −1 (−1,−1s) diffraction orders. The *color bar* shows extinction with *red* representing high extinction

The different morphological features of gratings are displayed in their optical characteristics. The extinction of incident P-polarized light in the gratings was estimated in the first approximation by the expression [16]: αd = ln((1-*R*)/*T*, where *R* and *T* are measured values of reflectance and transmittance. Obtained approximate values of αd as a function of wavelength and incidence angle are shown in Fig. 3 for gratings from Fig. 2. The samples were fixed in the rotary table of the measurement setup so that the plane of incidence was perpendicular to the grating grooves. Bands of high extinction are the result of a resonant excitation of surface plasmon-polaritons (SPPs) and localized plasmons (LP), mapping thereby the dispersion curves of the plasmon resonances.

Positions of SPP in these coordinates are calculated from the phase-matching condition [17, 18]:

1 $$ k\  \sin \theta + m G={k}_{\mathrm{PP}}, $$

where *k* = 2π/(*λ/n*) is the wave vector of the incident radiation with a wavelength λ in a vacuum; *θ* is the angle of incidence; *m* is an integer (*m* ≠ 0) and denotes the diffraction order; *G = 2*π*/a* is the reciprocal vector of grating with a period of *a*, *k*_PP_ is the wave vector of SPP.

The SPP wave vector is assumed to be the same as for a smooth metal interface [17]:

2 $$ {k}_{\mathrm{PP}}=\pm \left(2\uppi /\left(\uplambda / n\right)\right)\ {\left[{\upvarepsilon}_{\mathrm{Me}}\upvarepsilon /\left({\upvarepsilon}_{\mathrm{Me}}+\upvarepsilon \right)\right]}^{1/2}, $$

where *k*_PP_ have “+” sign at *m* > 0, and “−” at *m* < 0. Here, ε_Me_ = e’_Me_ + *i*e”_Me_ = (*n*_*Me*_ *+ ik*_*Me*_)^2^ is the complex permittivity of the metal at the wavelength *λ*, ε *= n* ^*2*^ is the permittivity and refractive index of the air or glass substrate that depends on at which interface air/metal or metal/substrate the SPP is excited. For correct dispersion curve calculation, we have to take into account the size effect of electron effective mean free path reduction and corresponding Au optical constant changes upon the formation of nanostructured relief but it is quite complicated. We believe that for identification of the type of mode this approximation is quite reasonable.

The spectral-angular position of SPP resonances were calculated by using the expressions (1–2) and are shown by solid lines on Fig. 3. For calculations, the optical constants of gold from ref. [19] were used, and the refractive index of the substrate was taken equal to *n* = 1.48. For a given grating period and optical constants, the excitation of modes with *m* = +1 (1,1s) and *m* = −1 (−1,−1s) at the interface air/gold (1,−1) and gold/substrate (1s,−1s) are possible.

It is seen that in gratings with small modulation depth (Fig. 3a, b) only two SPP modes with *m* = −1 (−1 and −1s) are observed. With increase of groove depth, the intensity of SPP decreases and localized plasmon resonance with band maximum near 683 nm at 10° incident angle appears (Fig. 3c). This LP resonance is more intense at small incident angles and overlaps with the −1s SPP mode. For grating with isolated grooves (nanowires), LP resonance is mostly observed with maximum at 570 nm (Fig. 3d), but the shape of the LP peak is slightly deformed due to the LP–SPP interaction. The peculiarity of LP excitation in a periodic array of nanowires is a weak dependence of the LP resonance spectral position on the angle of incidence [12] which is mostly due to angular dependence of the width of nanowire projection in the plane of light wave vector.

After additional deposition of gold onto grating with isolated grooves, the intensity of the SPP mode increases again and the LP band maximum shifts to longer wavelengths from 570 nm for the initial grating (Fig. 3d) to 920 nm after deposition of 10 nm (Fig. 3e) and to 980 nm after deposition of 20 nm (Fig. 3f).

To estimate the spectral position of the LP resonance, we can use the formula for the polarization of the spherical metal nanoparticle in dielectric environment [20], which in the case of small gold nanoparticle in a vacuum reaches a maximum in the vicinity of *λ ~* 0.5 microns. The resonant wavelength can significantly increase [21] and reach the infrared region of the spectrum, when the nanoparticle size (width of nanowires) and/or dielectric constant of the environment is increased; it is also increased with decreasing the distance between the nanoparticles/nanowires or in the presence of metal interlayer between nanowires (as in our case).

Thus, we observed a smooth transition from the SPP to LP excitation in case of an increase in the modulation depth of the grating. The appearance of resonance of localized plasmons is observed for our gratings, when the metal thickness between the grating grooves is reduced to 10 nm or less (Fig. 3c). Pure localized plasmons are observed in grating with isolated grooves (Fig. 3d), when slits between grooves are wider than half-period of the grating (actual for our case). We observed again the mixed mode with excitation of both SPP and LP after additional deposition of gold onto such grating (Fig. 3e, f).

These data are consistent with the results of ref. [14], where thin gold nanoslit gratings with variable slits are investigated experimentally and numerically. The authors have identified the regimes of surface plasmon-polaritons, localized plasmons, and the mixed mode.

We have been carrying out similar measurements for S-polarized light, but no extrema in the extinction spectra related to the excitation of SPP or LP were observed.

## Conclusions

Using IL technology and vacuum chalcogenide photoresist, we fabricated a set of high-frequency Au gratings with different modulation depth and metal thickness of layer between the grating grooves. The Au gratings exhibit different optical responses due to the different types of plasmon modes they support. We have shown that there is a smooth transition in the optical response of these gratings from propagating surface plasmon modes to localized plasmon resonances with increasing of the modulation depth of the gratings and/or slit width between grating grooves. It means that the optical response of gratings can be adjusted in a wide spectral range by selecting their geometric parameters.

## Abbreviations

AFM: Atomic force microscope
IL: Interference lithography
LP: Localized plasmons
SPP: Surface plasmon-polaritons
